# Excitability Constraints on Voltage-Gated Sodium Channels

**DOI:** 10.1371/journal.pcbi.0030177

**Published:** 2007-09-21

**Authors:** Elaine Angelino, Michael P Brenner

**Affiliations:** 1 Systems Biology Graduate Program, Harvard University, Cambridge, Massachusetts, United States of America; 2 School of Engineering and Applied Science, Harvard University, Cambridge, Massachusetts, United States of America; Weizmann Institute of Science, Israel

## Abstract

We study how functional constraints bound and shape evolution through an analysis of mammalian voltage-gated sodium channels. The primary function of sodium channels is to allow the propagation of action potentials. Since Hodgkin and Huxley, mathematical models have suggested that sodium channel properties need to be tightly constrained for an action potential to propagate. There are nine mammalian genes encoding voltage-gated sodium channels, many of which are more than ≈90% identical by sequence. This sequence similarity presumably corresponds to similarity of function, consistent with the idea that these properties must be tightly constrained. However, the multiplicity of genes encoding sodium channels raises the question: why are there so many? We demonstrate that the simplest theoretical constraints bounding sodium channel diversity—the requirements of membrane excitability and the uniqueness of the resting potential—act directly on constraining sodium channel properties. We compare the predicted constraints with functional data on mammalian sodium channel properties collected from the literature, including 172 different sets of measurements from 40 publications, wild-type and mutant, under a variety of conditions. The data from all channel types, including mutants, obeys the excitability constraint; on the other hand, channels expressed in muscle tend to obey the constraint of a unique resting potential, while channels expressed in neuronal tissue do not. The excitability properties alone distinguish the nine sodium channels into four different groups that are consistent with phylogenetic analysis. Our calculations suggest interpretations for the functional differences between these groups.

## Author Summary

There are few quantitative examples for how functional constraints bound and shape evolution. Sodium channels are a central player in the propagation of action potentials. Action potentials fire above a critical voltage threshold. Below the voltage threshold the membrane potential recovers to a resting value, which is assumed to be unique. Here we ask whether the properties of mammalian voltage-gated sodium channels are determined by the simplest possible constraints. We demonstrate that the requirements, (1) a voltage threshold and (2) a unique resting potential, severely constrain sodium channel properties. These constraints contain no free parameters, depending only on the concentrations of potassium inside and outside the cell. We test these predictions on functional data from the nine mammalian genes encoding voltage-gated sodium channels. All measurements obey the excitability constraint, whereas channels expressed in the nervous system systematically violate the constraint for a unique resting potential. These properties alone distinguish the nine sodium channels into four groups consistent with phylogenetic analysis. Our calculations suggest that different channel types have evolved to perform different tasks.

## Introduction

Despite the relatively small number of genes in the human genome, there are many examples of groups of nearly identical genes that perform similar functions. Such diversity could either reflect redundancy or evolutionary specialization [1,2]. Specialization could result from tuning to different functional environments, or nearly identical genes might play very different functional roles [3].

Here we explore how functional constraints bound and shape evolution through an analysis of mammalian voltage-gated sodium channels. The primary function of voltage-gated sodium channels is to allow the propagation of action potentials [4]. Since Hodgkin and Huxley [5], mathematical models have suggested that sodium channel properties need to be tightly constrained for an action potential to propagate. In mammals, there are nine different genes encoding voltage-gated sodium channels [6], many of which are more than ≈90% identical by sequence [7]. On one hand, the sequence similarity of the channels presumably corresponds to similarity of their functional properties; this is consistent with the idea that these properties must be tightly constrained. On the other hand, the multiplicity of genes encoding sodium channels raises the question: why are so many different mechanisms for generating an action potential necessary? Sodium channels are predominantly found in specific anatomical regions, suggesting that they might be tuned for specific functions. For example, the channels Na_v_1.1, Na_v_1.2, Na_v_1.3, Na_v_1.6, and Na_v_1.7 are predominantly localized in the central and peripheral nervous systems; Na_v_1.8 and Na_v_1.9 primarily in the dorsal root ganglion; Na_v_1.4 primarily at skeletal muscular junctions; Na_v_1.5 primarily in cardiac tissue (Table 1) [8].

**Table 1:**
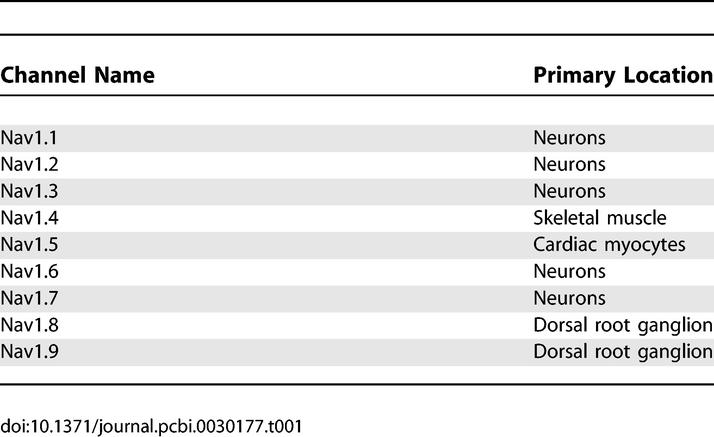
Primary Locations of Mammalian Voltage-Gated Sodium Channels Na_v_1.1–1.9

In this paper, we address the questions of whether and how sodium channel diversity is bounded by the simplest theoretical constraints on action potential propagation: (i) the sodium channel properties must be tuned to allow the membrane to be excitable, i.e., there must exist a voltage threshold above which an action potential can be produced, and (ii) the constraint of a unique resting potential. Through a theoretical analysis of macroscopic sodium currents, we demonstrate that these two requirements depend only on sodium channel properties, directly constraining the activation and inactivation curves of sodium channels, which are routinely directly measured in experiments. We then compare the constraints with measurements of mammalian sodium channels reported in the literature. Our dataset uses 172 different measurements from 40 distinct publications, including both wild-type and mutant Na_v_1.1–1.9, in human, mouse, and rat, under a range of different conditions including with and without different types of β subunits, and with chemicals (lidocaine, tetrodotoxin, etc.) [9–48]. The mutant channels tend to be associated with a disease state and hence presumably differ in a physiologically significant way from the wild-type.

Our analysis demonstrates that excitability properties alone distinguish the nine sodium channels into four different groups. Within each group there is a strong positive correlation between the voltage dependence of activation and inactivation. The members of each of the four groups are close according to phylogenetic analysis [7,49–53]. What are the functional differences between these groups? Two groups correspond to channels expressed in nerve and muscle tissue, respectively. Another group (consisting of the single channel Na_v_1.8) has the potential for a voltage threshold substantially higher than the other channels. The final group (consisting of the single channel Na_v_1.9) can only produce action potentials in a narrow conductance range and even then has a maximum voltage threshold which is less than thermal fluctuations. The separation of the channels into functionally distinct groups suggests that they have evolved to perform specialized tasks.

## Results

### Theoretical Constraints on Sodium Channel Properties

How are the properties of a voltage-gated sodium channel constrained by its function? The primary role of voltage-gated sodium channels is to make action potentials. Action potential generation corresponds to two fundamental requirements on the sodium channels. First, sodium channel properties must allow for the membrane to be excitable; namely there must exist a voltage threshold above which action potentials can be produced. Second, the sodium channel properties must give rise to a unique stable resting potential, where the sodium and potassium currents are in steady state. In the following we show that these criteria constrain sodium channel properties which are directly measured in routine experiments used to characterize sodium channels.

The basic model for an action potential was introduced by Hodgkin and Huxley [5]. The membrane potential *V* changes due to both sodium and potassium currents, so that

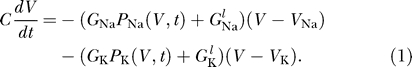
Here *G*_Na_ is the conductance of sodium due to voltage-dependent sodium channels, *P*_Na_ (*V*, *t*) is the time-dependent probability that a sodium channel is open, and 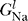 is the conductance of non-voltage-dependent sodium channels. The potassium channels are similarly characterized by *G*_K_, *P*_K_(*V*, *t*), and 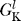 . The reversal potentials for sodium and potassium are fixed by the sodium and potassium concentrations on both sides of the membrane. The temporal dynamics of *P*_Na_(*V*, *t*) and *P*_K_(*V*, *t*) are determined by the kinetics of the sodium and potassium channels.

#### Excitability constraints depend only on sodium channel properties.

First we consider the constraints arising from excitability. The existence of a voltage threshold, above which an action potential can occur, can be analyzed by approximating Equation 1 as

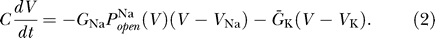
Here we have assumed (i) that the potassium channels open much more slowly than sodium channels [4] so that the potassium permeability is constant, and have written 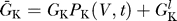 ; (ii) that the sodium leak conductance 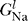 is much smaller than *G*_Na_ [5]; and (iii) that the probability that a sodium channel is open is given by some voltage-dependent function, 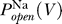 .

Given these assumptions, we now ask, under what conditions is the membrane excitable? In particular, for a sodium channel whose open probability is characterized by the the function 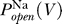 , what are the requirements for excitability? To analyze this, note that excitability requires that there are multiple fixed points of Equation 2. These fixed points obey

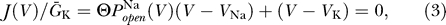
where *J*(*V*) is the current across the membrane, and we have divided by the conductance of the potassium channels *¯*_K_ to emphasize that the fixed points depend on both the properties of the sodium channel represented by the open probability 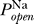 (*V*) and the dimensionless parameter 
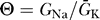
 . The value of Θ is not known in general; it is under the control of the cell and is determined by the relative expression levels of sodium and potassium channels.

Excitability requires multiple solutions to Equation 3. One such solution is the (stable) resting potential, and another solution is the unstable state whose distance from the resting potential determines the voltage threshold. We thus ask, does there exist a range of Θ for which there are multiple fixed points? This is determined by the functional form of the sodium channel's open probability 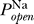 (*V*), and in particular does not depend on the number densities of sodium or potassium channels given by the conductances *G*_Na_ and *¯*_K_, respectively. Thus, excitability constraints depend only on sodium channel properties, specifically those given by the open probability 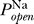 (*V*) .

#### Uniqueness of the resting potential depends only on sodium channel properties.

We now demonstrate that the requirement that there is a unique resting potential also imposes a constraint on sodium channel properties. The resting potential is determined by Equation 1 at steady state, i.e.,

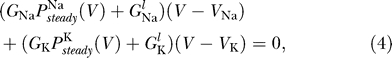
where 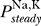 denote the steady state open probabilities of the sodium and potassium channels, respectively. The voltage-dependent potassium channels are mainly closed at the equilibrium potential, so we can therefore neglect *G*_K_ 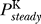 .

The requirement that there is a unique value of the resting potential thus translates into the requirement that there is only a single solution to the equation

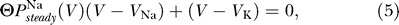
for any value of 
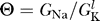
 . Remarkably, this constraint is precisely the opposite of the excitability constraint derived above, i.e., we require that there is a range of Θ for which there is a single fixed point to this equation and no regime of Θ where there are multiple fixed points. As in the case of the excitability constraint, whether this criterion is satisfied is determined by the functional form of 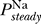 (*V*). Thus, uniqueness of the resting potential depends only on sodium channel properties, specifically those given by the steady state open probability 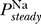 (*V*).

#### Functional form of *P_open_*(*V*) and *P_steady_*(*V*).

We have thus demonstrated that the excitability constraint and uniqueness of the resting potential present essentially the same mathematical problem and depend only on the sodium channel properties represented by the probability of a channel opening *P_open_*(*V*) and the steady state open probability of a channel *P_steady_*(*V*), respectively. To make further progress we need to know the functional forms of *P_open_*(*V*) and *P_steady_*(*V*).

**Figure 1:**
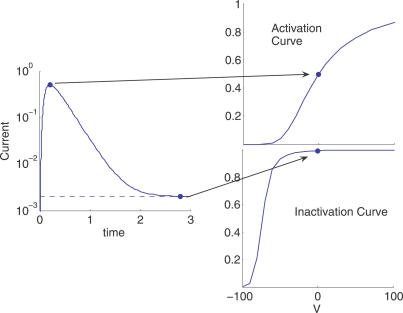
Experimental Characterization of Sodium Channels Used in This Paper A step change in the membrane potential from a very negative value (for *t* < 0) where all the channels are closed, to *V* (at *t* = 0) results in a measurable current. The left panel shows the current for *V* = 0 mV. The activation curve (upper right) is constructed by plotting the maximum current as a function of *V*, normalized by the maximum current as *V* → ∞. The inactivation curve (lower right) is the steady state current as a function of *V*, also normalized by the steady state value as *V* → ∞. This figure was produced using Kuo and Bean's model for sodium channels [56].

Both of these quantities are measured with a common experimental protocol, shown in Figure 1. For time *t* < 0, the membrane potential is initially fixed to be sufficiently negative so that all of the channels are in their closed state. At time *t* = 0, the membrane potential is then instantaneously stepped to some higher voltage *V*, leading to a measurable current. The current reaches a (*V* dependent) maximum value, and then settles down to a (*V* dependent) steady state value. The left panel in Figure 1 shows a typical current trace. The maximum current that is achieved corresponds to the probability of a channel opening and is called the activation curve, *P* ^act^(*V*). The approximation that *P* ^act^(*V*) gives the open probability *P_open_*(*V*) in Equation 2 can be demonstrated directly from the full kinetics of the channel assuming that the channels open sufficiently quickly [54]. The steady state current is called the inactivation curve, *P* ^inact^(*V*), and corresponds to the steady state open probability *P_steady_*(*V*). The activation and inactivation curves are both *V* dependent and normalized by the maximum current observed as *V* → ∞. It is well accepted in the literature (and confirmed by our own fits, see Materials and Methods) that both activation and inactivation curves are well fit by Boltzmann functions of the form

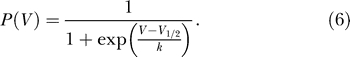



The Boltzmann functions corresponding to the activation and inactivation curves *P* ^act^(*V*) and *P* ^inact^(*V*) each depend on two parameters, *V*_1/2_ and *k*, that both have units of voltage. Combining this with the results of the last section then implies that the excitability constraint depends only on the sodium channel properties represented by the activation curve parameters 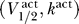 , and the uniqueness of the resting potential depends only on the sodium channel properties represented by the inactivation curve parameters 
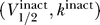
 .

#### Constraints on sodium channel properties.

Since both the activation and inactivation curves are given by Boltzmann functions as shown in Equation 6, the derivations of the excitability constraint and criterion for the uniqueness of the resting potential correspond to complementary sides of the same mathematical problem.

**Figure 2:**
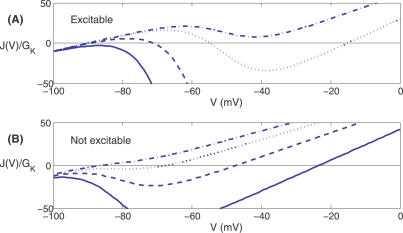
Current *J*(*V*/ ¯_K_ Given by Equation 3 for Different Values of 
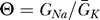 (A) The sodium channel is characterized by (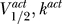)=(−50 mV, 6 mV), and the dot-dashed, dotted, dashed, and solid lines correspond to Θ = 0.5, 1, 5, 20. For Θ = 1, 5, there are multiple fixed points (i.e., zero crossings), and hence this channel is excitable. (B) The sodium channel is characterized by (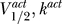)=(−80 mV, 6 mV), and the dot-dashed, dotted, dashed, and solid lines correspond to Θ = 0.1, 0.2, 0.4, 0.8. There is a single fixed point for each, and hence for this channel excitability is impossible.

We first consider the excitability constraint on the activation curve. Figure 2 demonstrates that whether a sodium channel is excitable depends critically on its 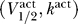 . Figure 2A shows the membrane current normalized by the potassium conductance, 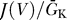 , as in Equation 3, for a sodium channel with 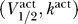 = (−50 mV, 6 mV) and Θ = 0.5, 1, 5, 20 (dot-dashed, dotted, dashed, and solid lines, respectively), where we take *V*_Na_ = 60 mV and *V*_K_ = −90 mV [4]. There are multiple fixed points for Θ = 1 and 5, so this channel can cause excitability for these parameter values. For an excitable channel, there is in general a range Θ*_min_* ≤ Θ ≤ Θ*_max_* where there are multiple fixed points to Equation 3. In contrast, Figure 2B shows a channel for 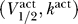 = (−80 mV, 6 mV), with Θ = 0.1, 0.2, 0.4, 0.8 (solid, dashed, dotted, and dot-dashed lines, respectively). Here for every Θ there is a single fixed point. Hence for a channel with 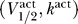 = (−80 mV, 6 mV), excitability is impossible.

**Figure 3:**
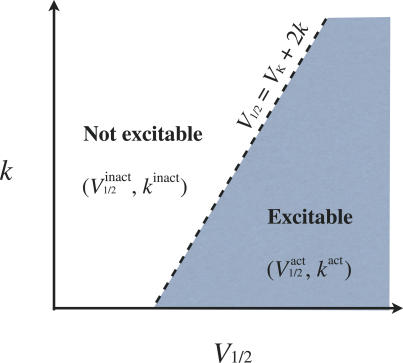
Schematic Showing Projection of Activation and Inactivation Curve Parameters onto the Same Two-Dimensional Space (*V*_1/2_, *k*) The activation 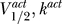
and inactivation 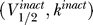 curve parameters are predicted to lie on opposite sides of the excitability threshold given by the line *V*_1/2_ = *V*_K_ + 2*k*.

We have carried out a mathematical analysis that follows from the excitability constraint given by Equation 2 to determine for which 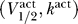 a channel is excitable (see Materials and Methods). The result is that a channel is excitable if and only if the following inequality is satisfied:

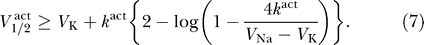
Since 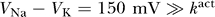 , this equation simplifies to

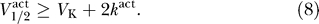
Solving for the criterion for the uniqueness of the resting potential follows an analogous mathematical analysis, yielding for 
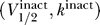
 the opposite result of the excitability constraint. The above Equation 8 then immediately implies that the uniqueness of the resting potential requires that

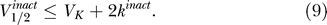
Thus, if we project the activation and inactivation curve parameters 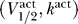 and 
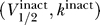
 , respectively, onto the same two-dimensional space (*V*_1/2_, *k*), then they lie on opposite sides of the excitability threshold given by the line *V*_1/2_ = *V*_K_ + 2*k*. A schematic of this prediction is shown in Figure 3. It is particularly noteworthy that the derivation of these results depends only on the sodium channel properties represented by the activation and inactivation curve parameters, and in particular is independent of the unknown conductance parameters *G*_Na_ and *¯*_K_. It is worth emphasizing that the predicted constraints on sodium channels do however depend critically on the reversal potential for *potassium* ions *V*_K_. For mammalian cells, the intracellular versus extracellular potassium concentrations predict an equilibrium of *V*_K_ ≈ −90 mV [4].

### Comparison of Measured Sodium Channel Properties to Constraints

The predicted constraints on sodium channels can be directly compared with measured sodium channel properties. We have collected activation and inactivation curve data of the various sodium channels from papers in the recent literature. The dataset includes papers where (*V*_1/2_, *k*) are reported explicitly, and also includes data that we digitized directly from the literature. Since each activation and inactivation curve is represented by two parameters (*V*_1/2_, *k*), we can represent the corresponding property space in two dimensions.

**Figure 4:**
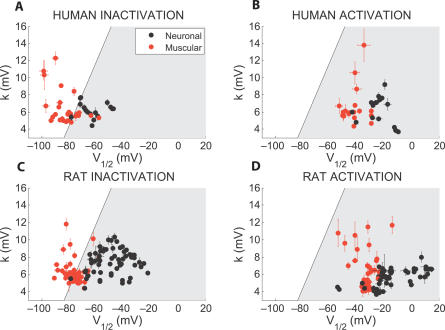
Summary of Activation and Inactivation Data for Human and Rat Voltage-Gated Sodium Channels The left plots (A) and (C) show inactivation data for human and rat, while the right plots (B) and (D) show activation data for human and rat. The black symbols represent the neuronal channels (Na_v_1.1, 1.2, 1.3, 1.6, 1.7, 1.8, 1.9) and the red symbols represent the muscular channels (Na_v_1.4, 1.5). The solid line is the excitability threshold. The activation data is predicted to lie in the shaded region.

Figure 4 shows this for humans (A,B) and rats (C,D), respectively. The left half of Figure 4A and 4C represents the inactivation curves, while the right half of Figure 4B and 4D shows the data for the activation curves. The black symbols represent the neuronal channels (Na_v_1.1, 1.2, 1.3, 1.6, 1.7, 1.8, 1.9) and the red symbols represent the muscular channels (Na_v_1.4, 1.5). For each channel type we include measurements for both wild-type and mutant, as well as a range of conditions: these include with or without subunits; with or without external effectors such as calmodulin, etc. Dataset S1 summarizes all of the collected data, including specific references, mutations, and conditions.

Figure 4A–4D represents the excitability threshold as a solid line. To the right of the solid line, excitability is possible, and to the left it is impossible. Consistent with our analysis of the excitability constraints, all measured activation curves 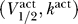 are on the right side of the excitability threshold.

On the other hand, the inactivation curves do not all obey the constraint given by the uniqueness of the resting potential. For the assumed *V*_K_ = −90 mV, we find that the channels expressed in muscle (Na_v_1.4, Na_v_1.5) obey the constraint whereas the neuronal channels do not. The implication of this is that for the neuronal channels, there exists a range of conductances (*G*_K_, *G*_Na_) for which there are multiple fixed points. We cannot say whether there is physiological significance to this parameter regime but note that an unstable resting potential might be advantageous for spontaneous firing.

**Figure 5:**
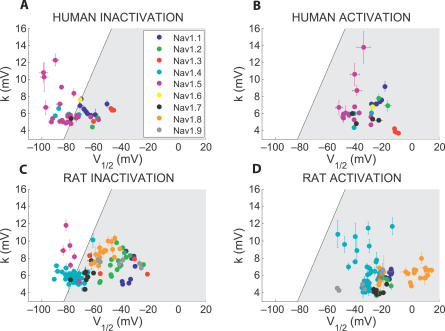
Summary of Activation and Inactivation Data for Human and Rat Voltage-Gated Sodium Channels The left plots (A) and (C) show inactivation data for human and rat. The right plots (B) and (D) show activation data for human and rat. The different colors represent different channel types, with blue, green, red, cyan, magenta, yellow, black, orange, grey representing Na_v_1.1, 1.2, 1.3, 1.4, 1.5, 1.6, 1.7, 1.8, 1.9, respectively. The solid line is the excitability threshold. The activation data is predicted to lie in the shaded region.

Figure 5 shows this same data for humans (A,B) and rats (C,D), respectively. Now we use different colors for each channel type, with blue, green, red, cyan, magenta, yellow, black, orange, grey representing Na_v_1.1, 1.2, 1.3, 1.4, 1.5., 1.6, 1.7, 1.8, 1.9, respectively.

#### Voltage threshold constraints.

We now extend the excitability argument one step further, and demonstrate that if 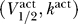 are in the excitable regime given by Equation 8, the specific values of 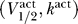 that characterize a sodium channel determine two other important properties of action potential physiology: (1) the maximum voltage threshold that is possible, where the voltage threshold is defined to be the distance between the resting potential (the fixed point at *V* ≈ *V*_K_) and the nearest additional fixed point, and (2) the range of Θ where excitability occurs. Both of these properties are physiologically important: for reliable action potential firing, we need to require that the voltage threshold be larger than the voltage fluctuations produced by thermal fluctuations, which are of order *k_B_*T/*e*. The smaller the range of Θ for which the membrane is excitable, the more difficult it is for the cell to tune channel properties to this region.

For each 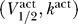 , the maximum voltage threshold occurs when Θ = Θ*_min_*. This can be seen in Figure 2A, where the voltage threshold for Θ = 1 is larger than that for Θ = 5. The fact that the maximum voltage threshold occurs at Θ*_min_* can be seen from noting that when Θ = 0 ≤ Θ < Θ*_min_*, the single equilibrium potential is *V* = *V*_K_. At the bifurcation point Θ = Θ*_min_*, an additional solution is created. As Θ continues to increase, two solutions are created, one of which eventually coalesces with the solution near V_K_. Finally, as Θ → ∞, there is only a single solution at *V* = *V*_Na_. The largest voltage threshold, defined as the distance between the equilibrium point near *V* = *V*_K_ and the closest other equilibrium point, therefore occurs at Θ = Θ*_min_*.

**Figure 6:**
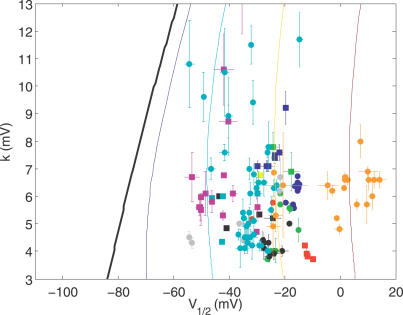
Activation Data for Human (Squares) and Rat (Circles), for Different Channel Types and Conditions As above, the different colors represent different channel types, with blue, green, red, cyan, magenta, yellow, black, orange, grey representing Na_v_1.1, 1.2, 1.3, 1.4, 1.5, 1.6, 1.7, 1.8, 1.9, respectively. The thick black line is the excitability threshold; the thin dark blue, light blue, orange, and red lines represent voltage thresholds of *k_B_*T/*e*, 2*k_B_*T/*e*, 3*k_B_*T/*e*, and 4*k_B_*T/*e*, respectively.

We can use this fact to explicitly compute the maximum voltage threshold as a function of 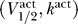 . Figure 6 shows a contour plot of the voltage thresholds, compared against activation data for human (squares) and rat (circles), in a variety of different conditions. The thick blue line represents the excitability threshold; the thin dark blue, light blue, orange, and red lines are the contours where the maximum voltage thresholds are *k_B_*T/*e*, 2*k_B_*T/*e*, 3*k_B_*T/*e*, and 4*k_B_*T/*e*, respectively. If we let Γ be the distance between 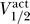 and the excitability boundary from Equation 8, then channels with a maximum voltage threshold of *k_B_*T/*e*, 2*k_B_*T/*e*, 3*k_B_*T/*e* satisfy

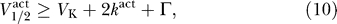
with Γ ≈ 15, 40, 60 mV, respectively.

The cardiac channels Na_v_1.5 (magenta symbols) have the lowest voltage thresholds at around 2*k_B_*T/*e*, with Γ ≤ 40 mV. Skeletal muscular channels Na_v_1.4 (cyan symbols) also have predicted maximum thresholds in this range (∼2*k_B_*T/*e*). On the other hand, most neuronal channels have Γ ∼ 60 mV and thus maximum voltage thresholds which are in the 3*k_B_*T/*e* range. The highest threshold voltage channels are Na_v_1.8 (orange symbols), with Γ ≈ 80 mV, corresponding to a voltage threshold of about 4*k_B_*T/*e*.

What about the range of Θ where excitability is possible? As Θ increases from Θ*_min_* to Θ*_max_*, the voltage threshold decreases from its maximum value to zero. Hence, when the maximum voltage threshold is very small, the range of Θ where excitability is possible is small; when the maximum voltage threshold is high, there is a wider range of Θ where excitability is possible.

#### The relationship between 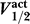 and 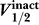 .

**Figure 7:**
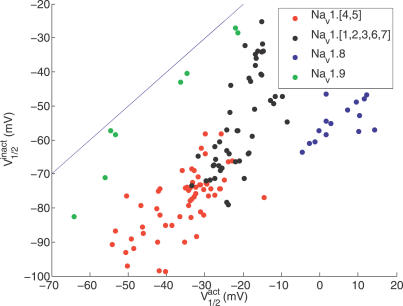
Correlation between 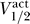 and 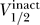 for Channels Colored by Four Different Groups The four groups are: (i) the non-muscular channels (Na_v_1.1, 1.2, 1.3, 1.6, 1.7) (black), (ii) muscular channels (Na_v_1.4, 1.5) (red), (iii) the channel Na_v_1.8 (blue), and (iv) the channel Na_v_1.9 (green). The solid line shows 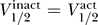 . This plot contains all data for which we have measurements of both inactivation and activation properties, including wild-type, mutant, and different conditions for human, rat, and mouse.

The data presented so far demonstrates systematic differences in (*V*_1/2_, *k*) for activation and inactivation curves between the different channel types, in humans and rats. Figure 7 replots this data from a different point of view, showing 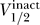 as a function of 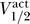 . Figure 7 contains all of the data for which we have measurements of both inactivation and activation curves, including wild-type, mutant, and different conditions for human, rat, and mouse.

In this representation it is clear that the channels break into four different groups: (i) the channels Na_v_1.1, 1.2, 1.3, 1.6, 1.7, which are primarily expressed in nervous tissue (black); (ii) those expressed primarily in muscle, Na_v_1.4, 1.5 (red); (iii) the channel Na_v_1.8 (blue); and (iv) the channel Na_v_1.9 (green).

This grouping of the channels has been previously observed in phylogenetic analyses. Plummer and Meisler [51] observed that by far the most confident branch point in their phylogenetic tree differentiated between Na_v_1.9 and the other channels Na_v_1.1, 1.2, 1.3, 1.4, 1.5, 1.6, 1.7, 1.8. The second most confident branch differentiated Na_v_1.8 from the other channels. Finally, it has been noted that the channels Na_v_1.1, 1.2, 1.3, 1.6, 1.7, which are expressed in the central nervous system, are more similar to each other than to the channels Na_v_1.4, 1.5 expressed in muscle [50].

Strikingly, within each group there is a strong correlation between 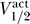 and 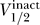 , which is reasonably approximated by a linear relationship with a slope of unity:

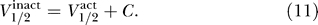



The offset parameter *C* changes between the different groups. The first two groups (i) and (ii) from above lie on the same line with *C* = −40 ± 10 mV, whereas Na_v_1.8 has *C* ≈ −60 ± 10 mV. In contrast, the data for Na_v_1.9 lie very close to the line 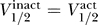 , with *C* ≤ 5 ± 5 mV.

**Figure 8:**
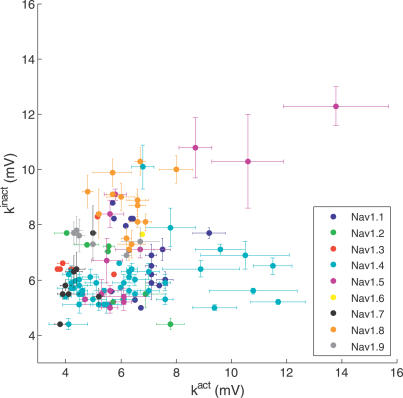
Correlation between *k* ^act^ and *k* ^inact^ As above, the different colors represent different channel types, with blue, green, red, cyan, magenta, yellow, black, orange, grey representing Na_v_1.1, 1.2, 1.3, 1.4, 1.5, 1.6, 1.7, 1.8, 1.9, respectively. This plot contains all data for which we have measurements of both inactivation and activation properties, including wild-type, mutant, and different conditions for human, rat, and mouse.

One might imagine that the clustering observed in Figure 7 is a simple consequence of phylogenetic relatedness, without having an explicit relationship to functional constraints. Figure 8 shows *k* ^inact^ as a function of *k* ^act^, using the same dataset as Figure 7. It is apparent that there is no correlation between these parameters, hence suggesting that the relationship demonstrated in Figure 7 reflects functional constraints.

Indeed, we can understand the correlation between 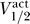 and 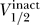 by using the relation from Equation 10, 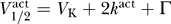 . Moreover, in general we expect that 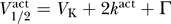 ; this is because (a) the resting potential is larger than *V*_K_, and (b) we expect that most of the channels are inactivated at the resting potential. These constraints lead to the inequality

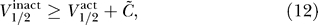
where *C̃* = −Γ − 2*k* ^act^. If we take Γ = 40, 60, 80 mV and assume that *k* ^act^ ≈ 6 mV, then we have *C̃* ≈ −52, −72, −92 mV, respectively. The data observe this constraint, with *C* ≥ *C̃* for each dataset.

It is worth remarking explicitly on channel Na_v_1.9, which has *C* ≤ −5 mV. According to our calculations, this implies that if Na_v_1.9 were to produce action potentials, the voltage threshold would be much less than *k_B_*T/*e*, i.e., smaller than the size of thermal fluctuations. As noted above, in this limit, the range of Θ where excitability is possible is small. This strongly implies that Na_v_1.9 is not used for producing action potentials. Recent studies characterizing Na_v_1.9 in dorsal root ganglion and sensory neurons where both Na_v_1.8 and Na_v_1.9 are expressed conclude that the Na_v_1.9 channels are not responsible for generating action potentials but instead are believed to generate depolarizations which help stimulate repetitive firing [33].

## Discussion

In this paper, we have considered the functional diversity of mammalian voltage-gated sodium channels as bounded by physical constraints on sodium channel function. We discussed two constraints: first, for a sodium channel to be able to generate an action potential it must be excitable, implying multiple equilibria for the current evoked by a step change in membrane potential; second, there is a constraint associated with a unique value of the resting potential in steady state. We showed that these constraints yielded results depending only on the sodium channel properties represented by the activation and inactivation curves, and collected corresponding data for human, mouse, and rat voltage-gated sodium channels, both wild-type and mutant, under a wide range of conditions. The excitability constraint was obeyed by all the data; on the other hand, the resting potential constraint was only obeyed in the channels expressed in muscle.

Furthermore, we demonstrated that there is a strong correlation between the voltage dependence of activation and inactivation in all of the channels. This correlation naturally breaks the channels into four different groups: (i) channels (Na_v_1.1, 1.2, 1.3, 1.6, 1.7), which are primarily expressed in nervous tissue, (ii) those expressed primarily in muscle (Na_v_1.4, 1.5), (iii) channel Na_v_1.8, and (iv) channel Na_v_1.9. The groups uncovered by analysis of this physiological data follow the major differences between channels shown by phylogeny. Most strikingly, the uniqueness of the physiological properties of Na_v_1.9 relative to the other channels is consistent with the phylogenetic assertion of Plummer and Meisler [51], who argue on the basis of sequence similarity that it evolved independently from the other channels. According to our analysis, it is essentially impossible for Na_v_1.9 to trigger action potentials.

In making all these conclusions, we have not distinguished between mutant and wild-type channels of a given type, despite the fact that the mutant channels included in our study are physiologically significant (generally leading to sodium channel disease). Additionally, we have not distinguished between the many different conditions in which the channels are expressed: our dataset (Dataset S1) includes channels with and without different types of subunits, and with chemicals (lidocaine, tetrodotoxin, etc.). The fact that the constraints and the correlations uncovered here hold so strongly indicates how robust they are. From this point of view, it is not surprising that our analysis has not completely provided a rationalization for all the channel diversity. In particular, we cannot distinguish the differences within either the group of muscular channels, Na_v_1.4, 1.5, or the group of neuronal channels, Na_v_1.1, 1.2, 1.3, 1.6, 1.7.

**Figure 9:**
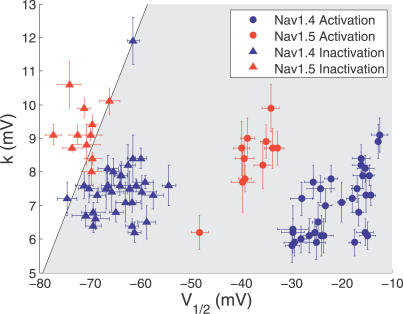
Activation (Circles) and Inactivation (Triangles) for Human Voltage-Gated Sodium Channels Na_v_1.4 (Blue) and Na_v_1.5 (Red) from [55] The solid line is the excitability threshold.

However, we believe there is significant opportunity for separating the channel properties further, even according to the admittedly crude metrics described here, if experiments were performed under more uniform conditions. An example of this is shown in Figure 9, compiled from a remarkably complete analysis [55] on the effects of calmodulin and other effectors on Na_v_1.4, 1.5. The triangle symbols of Figure 9 show 
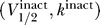
 while the circle symbols show 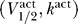 . The blue symbols correspond to Na_v_1.4 and the red to Na_v_1.5. This data shows a clear split between the properties of the two channels, which was not apparent from the data in Figures 5 and 7.

To these conclusions we need to add a strong caveat: we are not in any way suggesting that the physiological data analyzed here correspond to the most important differences between the different voltage-gated sodium channels. Indeed, it is clear that the kinetic properties of sodium channels are of critical importance for determining how they function. For example, repeated firing characteristics sensitively depend on channel properties and channel kinetics. Our analysis has not addressed the kinetic aspects of this problem at all, and we believe it is for this reason that our study has not been able to distinguish between mutant and wild-type channels of the same type.

Our omission of time constant information is not for lack of interest on our part, but instead because our review of the literature indicates that although a significant number of papers report measurements of both activation and inactivation curves, there are unfortunately far fewer consistent measurements of other channel properties that are critical for fully describing sodium channel function. These include the (voltage-dependent) timescales for activation and inactivation. Although many papers measure the inactivation timescales at positive voltages, a variety of different procedures are used for extracting these timescales from the raw data, and the raw data is not generally presented. This complicates comparing measurements with each other, and prevented our using them in this analysis.

We believe there is significant opportunity for extending the approach outlined here; namely, deriving constraints from simple models and comparing these results to kinetic properties of channel currents or even to single channels. There is little doubt that there are strong constraints on time constants for activation and inactivation in order for action potentials to fire properly. Uncovering these constraints (and hence the origin of the most critical differences between the channels) would lead to an understanding of the reasons for the differences between the different mammalian channels, and perhaps shed some light on how the channels contribute to nervous system function.

Ultimately such an approach could be applied to sodium, potassium, and calcium channels. There are no doubt constraints on sodium channels that arise from potassium channels and vice versa; for example, the time constant for inactivation of sodium channels must be tuned to the activation time constant for potassium channels for action potentials to fire properly. Repeated firing properties depend critically on the interaction of the various time constants. A complete and careful analysis of such constraints could be used as a tool to track and understand the evolution of the channels, perhaps relating to the origin of the nervous system itself. However, at present these ideas are at best immature speculation: for such studies to occur, it is necessary to expand efforts at acquiring kinetic properties of channels to non-mammalian species. There is much work to be done: our literature review was not able to uncover enough information about 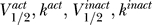 for invertebrate channels to include them in the present study.

## Materials and Methods

### Modeling the membrane potential.

Our theoretical analysis depended on a model for the membrane potential, given by Equation 1. We used the same model introduced by Hodgkin and Huxley [5], considering only contributions from sodium and potassium ions. Simplifying assumptions to this model are discussed in the text.

### Calculating the excitability threshold.

Here we calculate the excitability threshold. We are interested in characterizing the fixed points of the following equation:

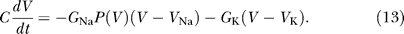
Setting *dV*/*dt* = 0 and rearranging gives that the fixed points obey

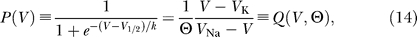
where we have made use of the measured functional form of the activation curve, *P*(*V*), given by Equation 6. The bifurcation points Θ = Θ_min,max_ are determined by looking for when the right and left hand sides of Equation 14 are tangent to each other,

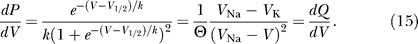



Equations 14 and 15 give two equations for the two unknowns (Θ, *V*), whose solutions give Θ*_min_* and Θ*_max_*, and the corresponding voltages at the bifurcation point. These equations can be solved by dividing Equation 14 by Equation 15. We obtain:

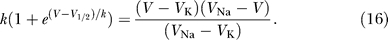



Combining the solution to Equation 16 for *V* with Equation 14 gives the bifurcation points Θ*_min_* and Θ*_max_*.

We are interested in computing the excitability threshold, namely the boundary in (*V*_1/2_, *k*) property space beyond which multiple solutions of Equation 14 do not exist for any values of Θ. This is guaranteed if there are *no* solutions to Equation 16, so that there is no range of Θ for which multiple intersections to Equation 14 exist. To identify the excitability threshold, we therefore find the (*V*_1/2_, *k*) where only a *single* solution to Equation 16 exists. This corresponds to requiring that the right-hand side of Equation 16 is tangent to the left-hand side. Differentiating the two sides gives

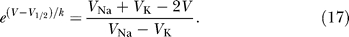



Combining Equations 16 and 17 implies that *V* = *V*_K_ + 2*k*. Inserting this into Equation 17 then implies that the excitability threshold is given by

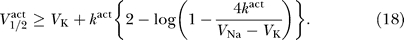



Now, since *V*_Na_ − *V*_K_ ≫ *k*, we can simplify Equation 18 to

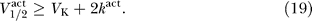



### Data collection.

To test our theoretical predictions on sodium channel properties, we collected activation and inactivation curves from papers in recent literature. The data is summarized in Dataset S1. The dataset includes papers where (*V*_1/2_, *k*) are reported explicitly, and also includes data that we digitized directly from the literature using the program GraphClick (Arizona Software, version 2.8.2). The values of (*V*_1/2_, *k*) from the digitized data were obtained by curve-fitting with Matlab.

## Supporting Information

Dataset S1 Voltage-Gated Sodium Channel Property Dataset (91 KB PDF)
