# Supplementary material for: Excitability Constraints on Voltage-Gated Sodium Channels
Source: PLoS Comput Biol. 2007 Sep 21;3(9):e177. doi: 10.1371/journal.pcbi.0030177 (PMC1988852; doi:10.1371/journal.pcbi.0030177)
Supplement: Supplementary file 1 [file pcbi.0030177.sd001.pdf]

Dataset S1

Here we include the dataset compiled for the analysis of this paper.

The first column lists the citation from which the data was extracted.

The second column lists the channel type $\mathrm{xNa_v1.y}$; the first variable 'x' is either 'r, h, m' for rat, human or mouse. The second variable 'y' is a number $1 \ldots 9$ denoting the specific channel type.

The third column lists whether the channel expressed is wild type or mutant. The mutant channels are characterized in the fashion they are described in the correspondingly cited paper: DX denotes that there is a mutation in domain 'X'; IFMQ3 denotes a mutation in the inactivation sequence; other notations specify the amino acid substitution directly.

The fourth column specifies the conditions under which the channel is expressed, as characterized in the correspondingly cited paper. The conditions include with or without β subunits, or other external conditions. It should not be assumed that the descriptor here is a full summary of the conditions under which the channel was expressed: for example unless we explicitly state that no β subunits are expressed, they may or may not be expressed.

Finally the next four columns denote the $V_{1/2}^{\mathrm{act}}, k^{\mathrm{act}}, V_{1/2}^{\mathrm{inact}}, k^{\mathrm{inact}}$. If the column is blank there was no measurement of this quantity reported.

We note that for publications [9-21], we digitized and fit the data to a Boltzmann curve ourselves; the error bars in these cases reflect 95% confidence intervals with respect to our fits. For the remaining publications [22-48] we report the fits (and error bars) given by the authors of the corresponding publication.

Citation	Channel	WT/mutant	Conditions	$V_{1/2}^{\text{act}}$ (mV)	k^{act} (mV)	$V_{1/2}^{\text{inact}}$ (mV)	k^{inact} (mV)
[1]	rNav1.2	WT	no β	—	—	-38.31 ± 0.04	8.08 ± 0.03
[1]	rNav1.2	WT	β1	—	—	-44.96 ± 0.08	6.68 ± 0.07
[1]	rNav1.2	WT	P0	—	—	-40.78 ± 0.06	9.22 ± 0.04
[1]	rNav1.2	WT	β1P0β1	—	—	-48.73 ± 0.06	7.86 ± 0.06
[1]	rNav1.2	WT	P0β1P0	—	—	-39.63 ± 0.05	8.61 ± 0.04
[1]	rNav1.2	WT	β1P0P0	—	—	-49.84 ± 0.06	7.18 ± 0.05
[1]	rNav1.2	WT	P0β1β1	—	—	-35.38 ± 0.04	8.31 ± 0.03
[2]	rNav1.1	WT	no β	-16.82 ± 0.21	5.66 ± 0.12	-34.93 ± 0.10	5.58 ± 0.08
[2]	rNav1.2	WT	no β	-18.38 ± 0.27	5.56 ± 0.20	-41.80 ± 0.14	7.22 ± 0.08
[2]	rNav1.1	WT	β1 + β2	-15.17 ± 0.08	6.50 ± 0.08	-40.96 ± 0.09	5.28 ± 0.08
[2]	rNav1.2	WT	β1 + β2	-21.60 ± 0.15	6.08 ± 0.10	-57.24 ± 0.13	5.29 ± 0.09
[3]	rNav1.1	WT	no β	-14.96 ± 0.14	6.30 ± 0.08	-31.75 ± 0.11	7.09 ± 0.07
[3]	rNav1.1	WT	β1	-14.7 ± 0.14	6.44 ± 0.09	-33.98 ± 0.13	8.22 ± 0.08
[3]	rNav1.1	DII	no β	-19.49 ± 0.11	5.76 ± 0.07	-32.37 ± 0.09	8.22 ± 0.09
[3]	rNav1.1	DII	β1	-15.17 ± 0.14	6.18 ± 0.09	-34.19 ± 0.12	7.96 ± 0.07
[3]	rNav1.1	DIV	no β	-16.87 ± 0.18	5.70 ± 0.09	-38.04 ± 0.10	8.79 ± 0.06
[3]	rNav1.1	DIV	β1	-16.23 ± 0.16	6.37 ± 0.10	-33.98 ± 0.13	8.22 ± 0.08
[4]	rNav1.2	IFMQ3	no β	-24.71 ± 0.07	4.01 ± 0.06	—	—
[4]	rNav1.2	IFMQ3	β1	-26.00 ± 0.06	3.71 ± 0.05	—	—
[4]	rNav1.3	WT	no β	—	—	-22.5 ± 0.08	6.15 ± 0.07
[4]	rNav1.3	WT	β1	—	—	-32.23 ± 0.10	7.92 ± 0.09
[5]	mNav1.6	WT	no β	-8.49 ± 0.14	6.70 ± 0.08	-54.72 ± 0.13	7.28 ± 0.08

Citation	Channel	WT/mutant	Conditions	$V_{1/2}^{\text{act}}$ (mV)	k^{act} (mV)	$V_{1/2}^{\text{inact}}$ (mV)	k^{inact} (mV)
[5]	mNav1.6	WT	$\beta1 + \beta2$	-17.05 ± 0.17	6.29 ± 0.10	-50.89 ± 0.07	4.76 ± 0.05
[5]	rNav1.1	WT	no β	-16.58 ± 0.18	5.56 ± 0.11	-35.96 ± 0.10	5.08 ± 0.06
[5]	rNav1.1	WT	$\beta1 + \beta2$	-15.36 ± 0.10	6.73 ± 0.09	-40.70 ± 0.08	5.00 ± 0.06
[5]	rNav1.2	WT	no β	-17.99 ± 0.20	5.52 ± 0.11	-42.71 ± 0.14	7.04 ± 0.10
[5]	rNav1.2	WT	$\beta1 + \beta2$	-21.42 ± 0.15	5.71 ± 0.1	-57.34 ± 0.14	5.21 ± 0.1
[6]	rNav1.2	WT	no β	-15.12 ± 0.34	4.77 ± 0.44	-25.28 ± 0.06	7.28 ± 0.06
[6]	rNav1.2	WT	$\beta1$	-23.19 ± 0.61	4.03 ± 0.43	-43.92 ± 0.08	7.69 ± 0.07
[7]	rNav1.3	WT	no β	-31.83 ± 0.09	5.15 ± 0.07	-64.9 ± 0.09	8.30 ± 0.08
[7]	rNav1.3	WT	$\beta3$	-24.20 ± 0.05	5.76 ± 0.04	-56.99 ± 0.07	6.19 ± 0.08
[8]	hNav1.6	WT	no β	-28.69 ± 0.10	6.77 ± 0.06	-71.87 ± 0.10	7.65 ± 0.06
[9]	hNav1.7	WT	$\beta1 + \beta2$	-28.94 ± 0.05	5.35 ± 0.04	—	—
[9]	hNav1.7	I848T	$\beta1 + \beta2$	-40.86 ± 0.05	4.83 ± 0.05	—	—
[9]	hNav1.7	L858H	$\beta1 + \beta2$	-43.63 ± 0.04	5.99 ± 0.04	—	—
[10]	hNav1.3	WT	no β	—	—	-61.99 ± 0.15	5.06 ± 0.07
[11]	hNav1.4	WT	no β	-42.49 ± 0.05	4.34 ± 0.05	-80.15 ± 0.08	5.96 ± 0.06
[11]	hNav1.4	R672G	no β	-42.33 ± 0.06	6.00 ± 0.05	-90.17 ± 0.07	5.73 ± 0.06
[11]	hNav1.4	R672H	no β	-45.99 ± 0.06	5.93 ± 0.05	-87.58 ± 0.07	6.60 ± 0.06
[12]	hNav1.5	WT	$\beta1$	-41.80 ± 0.05	6.77 ± 0.04	—	—
[12]	hNav1.5	MUTANT	$\beta1$	-50.25 ± 0.06	5.96 ± 0.04	—	—
[13]	mNav1.6	WT		-27.15 ± 0.06	7.06 ± 0.05	-65.71 ± 0.10	7.99 ± 0.08
[13]	mNav1.7	WT		-33.34 ± 0.09	6.79 ± 0.07	-73.53 ± 0.14	8.11 ± 0.12
[14]	hNav1.1	WT	$\beta1 + \beta2$	-26.4 ± 2.3	7.1 ± 0.2	-67.5 ± 2.3	6.2 ± 0.3

Citation	Channel	WT/mutant	Conditions	$V_{1/2}^{\mathrm{act}}$ (mV)	k^{act} (mV)	$V_{1/2}^{\mathrm{inact}}$ (mV)	k^{inact} (mV)
[14]	hNav1.1	R1648H	$\beta1 + \beta2$	-25.9 ± 1.0	7.1 ± 0.2	-69.1 ± 2.1	6.5 ± 0.4
[14]	hNav1.1	T875M	$\beta1 + \beta2$	-26.1 ± 1.2	7.1 ± 0.2	-60.7 ± 1.1	5.9 ± 0.6
[14]	hNav1.1	W1204R	$\beta1 + \beta2$	-29.6 ± 1.2	7.1 ± 0.2	-72.0 ± 2.0	6.9 ± 0.4
[15]	hNav1.1	WT	$\beta1 + \beta2$	-23.6 ± 1.2	7.4 ± 0.3	-64.2 ± 1.1	5.8 ± 0.1
[15]	hNav1.1	R1648C	$\beta1 + \beta2$	-19.7 ± 0.6	9.2 ± 0.6	-71.3 ± 0.2	7.7 ± 0.2
[15]	hNav1.1	F1661S	$\beta1 + \beta2$	-23.5 ± 0.4	7.5 ± 0.3	-52.0 ± 0.4	7.1 ± 0.4
[15]	hNav1.1	G1749E	$\beta1 + \beta2$	-22.0 ± 1.6	7.6 ± 0.3	-66.4 ± 1.1	6.0 ± 0.3
[16]	hNav1.5	WT	$\beta1$	-48.6 ± 1.2	6.1 ± 0.8	-92.0 ± 1.7	5.4 ± 0.3
[16]	hNav1.5	G514C	$\beta1$	-38.5 ± 1.4	6.1 ± 0.3	-85.1 ± 0.9	5.2 ± 0.3
[17]	rNav1.2	WT	no β	—	—	-45.1 ± 0.3	9.8 ± 0.2
[17]	rNav1.2	WT	$\beta1$	—	—	-49.1 ± 0.3	5.9 ± 0.2
[17]	rNav1.2	WT	$\beta3$	—	—	-49.3 ± 0.2	6.1 ± 0.1
[18]	hNav1.5	WT	no β	-50.1 ± 0.7	5.5 ± 0.6	-97.0 ± 2.3	6.7 ± 0.8
[18]	hNav1.5	WT	$\beta1$	-42.2 ± 0.8	5.3 ± 0.2	-75.0 ± 2.7	5.5 ± 0.5
[19]	hNav1.2	WT		-24.2 ± 2.0	7.8 ± 0.5	-63.1 ± 1.0	4.4 ± 0.2
[20]	rNav1.8	WT		-23.8	6.8	—	—
[21]	hNav1.5	WT		—	—	-78.6 ± 0.1	4.8 ± 0.1
[21]	hNav1.5	WT	cocaetylene (10 μM)	—	—	-80.8 ± 0.1	5.0 ± 0.1
[21]	hNav1.5	WT	cocaetylene (25 μM)	—	—	-82.1 ± 0.2	4.9 ± 0.1
[21]	hNav1.5	WT	cocaetylene (50 μM)	—	—	-84.1 ± 0.1	5.0 ± 0.1
[21]	hNav1.5	WT	cocaetylene (150 μM)	—	—	-86.7 ± 0.1	5.1 ± 0.1
[22]	rNav1.8	WT		10.3 ± 1.2	6.6 ± 0.4	-52.9 ± 0.8	8.9 ± 0.2

Citation	Channel	WT/mutant	Conditions	$V_{1/2}^{act}$ (mV)	k^{act} (mV)	$V_{1/2}^{inact}$ (mV)	k^{inact} (mV)
[22]	rNav1.8	WT	$\beta1$	1.6 ± 0.7	6.3 ± 0.1	-58.4 ± 1.5	7.1 ± 0.4
[22]	rNav1.8	WT	$\beta2$	9.5 ± 0.8	5.7 ± 0.7	-49.0 ± 0.2	9.9 ± 0.5
[22]	rNav1.8	WT	$\beta3$	11.5 ± 1.1	6.0 ± 0.5	-47.9 ± 0.6	9.0 ± 0.5
[22]	rNav1.8	WT	$\beta1 + \beta2$	1.7 ± 0.4	6.7 ± 0.2	-46.6 ± 0.9	10.3 ± 0.5
[22]	rNav1.8	WT	$\beta1 + \beta3$	2.9 ± 0.9	6.6 ± 0.2	-55.1 ± 0.9	8.7 ± 0.4
[22]	rNav1.8	WT	$\beta2 + \beta3$	7.2 ± 1.0	8.0 ± 0.6	-51.3 ± 1.2	10.0 ± 0.5
[22]	rNav1.8	WT	$\beta1 + \beta2 + \beta3$	1.7 ± 1.1	6.6 ± 0.3	-54.4 ± 1	8.9 ± 0.4
[23]	rNav1.7	WT	$\beta1$	-25.6 ± 1.5	3.8 ± 0.2	-68.4 ± 0.4	4.4 ± 0.1
[23]	rNav1.7	WT	$\beta1$, lidocaine (100 μM)	-23.9 ± 1.9	3.9 ± 0.2	-79.0 ± 1.5	5.5 ± 0.2
[23]	rNav1.8	WT	$\beta1$	6.2 ± 0.7	5.7 ± 0.2	-42.7 ± 1.2	9.1 ± 0.1
[23]	rNav1.8	WT	$\beta1$, lidocaine (100 μM)	12.3 ± 1.8	6.6 ± 0.3	-46.8 ± 0.6	8.1 ± 0.2
[24]	rNav1.7	WT	$\beta1$	-27.9 ± 0.8	4.4 ± 0.3	-67.7 ± 2.5	6.4 ± 0.6
[24]	rNav1.7	WT	$\beta1$, forskolin (1 μM)	-25.7 ± 0.4	4.3 ± 0.3	-68.3 ± 3.3	6.3 ± 0.6
[24]	rNav1.8	WT	$\beta1$	-1.2 ± 1.0	5.2 ± 0.1	-60.5 ± 2.7	8.4 ± 0.9
[24]	rNav1.8	WT	$\beta1$, forskolin (50 μM)	-0.3 ± 1.9	4.8 ± 0.2	-57.4 ± 2.3	9.2 ± 0.6
[24]	rNav1.7	WT	$\beta1$	-27.5 ± 1.3	4.1 ± 0.4	-67.3 ± 0.8	5.5 ± 0.2
[24]	rNav1.7	WT	$\beta1$, PMA (10 nM)	-21.0 ± 2.3	4.0 ± 0.2	-66.1 ± 1.4	5.8 ± 0.2
[24]	rNav1.7	WT	$\beta1$	-27.3 ± 0.6	4.3 ± 0.1	-61.9 ± 0.9	6.3 ± 0.2
[24]	rNav1.7	WT	$\beta1$, PDBu (20 nM)	-18.4 ± 0.8	5.0 ± 0.1	-63.8 ± 2.9	7.7 ± 1.0
[24]	rNav1.8	WT	$\beta1$	-4.6 ± 3.6	6.4 ± 0.5	-63.6 ± 1.4	7.3 ± 0.5
[24]	rNav1.8	WT	$\beta1$, PMA (2 nM)	10.1 ± 3.5	6.9 ± 0.2	-57.6 ± 3.0	8.1 ± 0.3
[24]	rNav1.8	WT	$\beta1$	-2.8 ± 1.2	6.2 ± 0.2	-61.1 ± 0.7	7.5 ± 0.4
[24]	rNav1.8	WT	$\beta1$, PDBu (2 nM)	14.3 ± 2.1	6.6 ± 0.3	-57.0 ± 1.2	8.7 ± 0.3

Citation	Channel	WT/mutant	Conditions	$V_{1/2}^{\text{act}}$ (mV)	k^{act} (mV)	$V_{1/2}^{\text{inact}}$ (mV)	k^{inact} (mV)
[25]	rNav1.9	WT	SNS, CsCl	-21.4 ± 0.8	6.7 ± 0.7	-28.5 ± 0.6	7.4 ± 0.5
[25]	rNav1.9	WT	SNS, CSF	-22.0 ± 0.3	6.2 ± 0.2	-27.1 ± 0.2	6.9 ± 0.2
[25]	rNav1.9	WT	NaN, CsCl	-36.3 ± 0.2	5.0 ± 0.1	-43.0 ± 0.5	7.3 ± 0.5
[25]	rNav1.9	WT	NaN, CsF	-53.3 ± 0.3	4.3 ± 0.2	-58.5 ± 0.4	7.7 ± 0.4
[25]	rNav1.9	WT	SNaC, CsCl	-34.6 ± 0.4	4.4 ± 0.2	-40.5 ± 0.5	7.8 ± 0.5
[25]	rNav1.9	WT	SNaC, CsF	-54.5 ± 0.3	4.5 ± 0.2	-57.2 ± 0.7	7.6 ± 0.6
[26]	hNav1.5	WT		-50.4 ± 1.3	5.6 ± 0.5	-76.4 ± 1.2	8.4 ± 0.5
[26]	hNav1.5	WT	FHF1B	-46.2 ± 1.1	5.8 ± 0.4	-85.7 ± 1.4	9.1 ± 0.2
[26]	hNav1.5	D1790G		-40.3 ± 3.2	8.7 ± 0.6	-98.7 ± 2.8	10.8 ± 1.1
[26]	hNav1.5	D1790G	FHF1B	-41.9 ± 3.5	10.6 ± 1.3	-98.4 ± 2.9	10.3 ± 1.7
[27]	hNav1.5	WT		—	—	-73	5.6
[27]	hNav1.5	WT	0.1 μM TTX	—	—	-74	5.8
[27]	hNav1.5	WT	3 μM TTX	—	—	-81	5.8
[27]	hNav1.5	WT	10 μM TTX	—	—	-79	5.8
[27]	hNav1.5	WT	30 μM TTX	—	—	-81	5.7
[28]	hNav1.5	WT		-50.6 ± 1.3	5.6 ± 0.3	-93.1 ± 1.7	5.0 ± 0.2
[28]	hNav1.4	WT		-29.6 ± 1.6	6.1 ± 0.4	-72.8 ± 2.1	5.9 ± 0.3
[28]	hNav1.2	WT		-17.6 ± 2.9	6.9 ± 0.7	-58.9 ± 1.7	5.5 ± 0.5
[28]	hNav1.7	WT		-24.3 ± 3.0	5.2 ± 0.2	-78.4 ± 1.0	5.4 ± 0.3
[29]	hNav1.5	WT	β	-29.8 ± 0.4	5.6 ± 0.4	-64.0 ± 0.8	5.6 ± 0.2
[29]	hNav1.5	L619F	β	-29.9 ± 0.4	4.7 ± 0.1	-58.2 ± 1.1	5.3 ± 0.2
[30]	rNav1.4	WT		-22.8 ± 0.9	6.4 ± 0.2	-66.1 ± 0.6	6.4 ± 0.2

Citation	Channel	WT/mutant	Conditions	$V_{1/2}^{\text{act}}$ (mV)	k^{act} (mV)	$V_{1/2}^{\text{inact}}$ (mV)	k^{inact} (mV)
[30]	rNav1.4	WT	$\beta 1$	-23.9 ± 1.2	6.3 ± 0.3	-66.5 ± 0.5	6.3 ± 0.2
[31]	rNav1.4	WT	V_{hold} = -60 mV	-25.7 ± 1.4	7.8 ± 0.9	-58.3 ± 1.7	7.9 ± 0.7
[31]	rNav1.4	WT	V_{hold} = -70 mV	-30.9 ± 1.0	6.8 ± 0.4	-62.4 ± 0.6	10.1 ± 0.8
[31]	rNav1.4	WT	V_{hold} = -85 mV	-32.8 ± 0.4	7.0 ± 0.4	-68.9 ± 0.7	6.3 ± 0.3
[31]	rNav1.4	WT	V_{hold} = -110 mV	-41.7 ± 1.9	7.6 ± 0.3	-74.3 ± 0.8	5.3 ± 0.2
[31]	rNav1.4	WT	V_{hold} = -130 mV	-46.6 ± 1.6	7.0 ± 0.4	-79.2 ± 1.0	5.6 ± 0.2
[32]	rNav1.4	WT		—	—	-72.6 ± 1.0	6.2 ± 0.4
[32]	rNav1.4	WT	steroid-treated	—	—	-72.4 ± 1.2	5.7 ± 1.0
[32]	rNav1.4	WT	denervated	—	—	-76.4 ± 1.2	6.5 ± 0.4
[32]	rNav1.4	WT	steroid-denervated	—	—	-80.0 ± 1.1	5.0 ± 0.9
[32]	rNav1.5	WT	denervated	—	—	-79.3 ± 1.6	9.5 ± 0.7
[32]	rNav1.5	WT	steroid-denervated	—	—	-82.3 ± 1.1	11.8 ± 0.7
[33]	hNav1.3	12v1		-12.1 ± 0.9	3.9 ± 0.2	-49.4 ± 0.3	6.6
[33]	hNav1.3	12v2		-12.8 ± 0.7	4.2 ± 0.1	-48.5 ± 0.5	6.4
[33]	hNav1.3	12v3		-11.7 ± 0.7	3.8 ± 0.1	-47.3 ± 0.5	6.4
[33]	hNav1.3	12v4		-9.8 ± 0.6	3.7 ± 0.1	-47.3 ± 0.3	6.4
[34]	rNav1.4	WT	$\beta 1$	-32.7 ± 1.0	5.4 ± 0.2	-76.8 ± 0.7	5.6 ± 0.1
[34]	rNav1.4	I424C	$\beta 1$	-31.2 ± 1.5	4.6 ± 0.4	-73.1 ± 1.3	5.5 ± 0.3
[34]	rNav1.4	I425C	$\beta 1$	-26.1 ± 0.7	7.6 ± 0.2	-74.4 ± 0.6	5.9 ± 0.3
[34]	rNav1.4	F426C	$\beta 1$	-33.6 ± 1.7	4.9 ± 0.3	-82.8 ± 1.2	6.1 ± 0.3
[34]	rNav1.4	L427C	$\beta 1$	-34.6 ± 1.0	5.2 ± 0.3	-74.0 ± 1.8	5.1 ± 0.2
[34]	rNav1.4	G428C	$\beta 1$	-32.3 ± 1.0	6.1 ± 0.4	-75.9 ± 0.6	5.3 ± 0.1
[34]	rNav1.4	S429C	$\beta 1$	-32.4 ± 2.6	4.5 ± 0.3	-74.0 ± 1.1	5.8 ± 0.4

Citation	Channel	WT/mutant	Conditions	$V_{1/2}^{\mathrm{act}}$ (mV)	k^{act} (mV)	$V_{1/2}^{\mathrm{inact}}$ (mV)	k^{inact} (mV)
[34]	rNav1.4	F430C	$\beta1$	-31.2 ± 1.7	5.1 ± 0.4	-81.2 ± 1.1	5.9 ± 0.3
[34]	rNav1.4	Y431C	$\beta1$	-31.8 ± 1.4	4.2 ± 0.5	-70.8 ± 1.1	5.7 ± 0.1
[34]	rNav1.4	L432C	$\beta1$	-29.5 ± 1.7	6.5 ± 0.4	-73.0 ± 0.9	6.0 ± 0.2
[34]	rNav1.4	I433C	$\beta1$	-30.2 ± 1.2	6.3 ± 0.4	-74.7 ± 1.2	5.6 ± 0.2
[34]	rNav1.4	N434C	$\beta1$	-30.2 ± 1.1	6.3 ± 0.2	-82.0 ± 0.7	6.1 ± 0.2
[34]	rNav1.4	L435C	$\beta1$	-33.9 ± 1.6	4.5 ± 0.3	-77.8 ± 1.7	5.8 ± 0.3
[34]	rNav1.4	I436C	$\beta1$	-24.9 ± 1.2	5.5 ± 0.5	-71.9 ± 0.7	5.1 ± 0.3
[34]	rNav1.4	V779C	$\beta1$	-36.6 ± 1.1	4.6 ± 0.2	-82.5 ± 1.5	5.6 ± 0.1
[34]	rNav1.4	M780C	$\beta1$	-29.1 ± 2.0	4.2 ± 0.2	-76.5 ± 1.9	6.3 ± 0.2
[34]	rNav1.4	V781C	$\beta1$	-34.3 ± 1.9	4.5 ± 0.3	-74.9 ± 1.8	5.5 ± 0.3
[34]	rNav1.4	I782C	$\beta1$	-33.5 ± 2.0	4.5 ± 0.6	-68.6 ± 0.6	5.1 ± 0.3
[34]	rNav1.4	G783C	$\beta1$	-34.2 ± 1.4	4.1 ± 0.2	-79.4 ± 2.7	6.2 ± 0.2
[34]	rNav1.4	N784C	$\beta1$	-33.8 ± 1.0	4.5 ± 0.4	-74.1 ± 1.1	6.0 ± 0.3
[34]	rNav1.4	L785C	$\beta1$	-26.0 ± 1.4	4.0 ± 0.2	-74.3 ± 0.8	5.4 ± 0.1
[34]	rNav1.4	V786C	$\beta1$	-35.9 ± 1.6	4.1 ± 0.7	-69.1 ± 1.6	4.4 ± 0.2
[34]	rNav1.4	V787C	$\beta1$	-27.3 ± 1.2	6.5 ± 0.5	-76.3 ± 0.6	5.5 ± 0.1
[34]	rNav1.4	L788C	$\beta1$	-33.4 ± 1.8	4.3 ± 0.3	-77.4 ± 0.8	5.7 ± 0.2
[34]	rNav1.4	N789C	$\beta1$	-28.5 ± 1.0	4.2 ± 0.2	-74.3 ± 0.9	5.9 ± 0.2
[34]	rNav1.4	L790C	$\beta1$	-33.1 ± 1.7	5.0 ± 0.5	-72.6 ± 1.0	5.3 ± 0.2
[34]	rNav1.4	F791C	$\beta1$	-35.0 ± 0.9	5.4 ± 0.5	-74.1 ± 1.0	5.1 ± 0.1
[35]	rNav1.4	WT		-32.1 ± 0.8	11.5 ± 0.7	-88.5 ± 2.4	6.5 ± 0.3
[35]	rNav1.4	I687M		-49.2 ± 1.0	9.6 ± 0.9	-89.2 ± 2.1	7.1 ± 0.2

Citation	Channel	WT/mutant	Conditions	$V_{1/2}^{\text{act}}$ (mV)	k^{act} (mV)	$V_{1/2}^{\text{inact}}$ (mV)	k^{inact} (mV)
[35]	rNav1.4	I689T		-41.6 ± 1.7	10.5 ± 1.6	-82.1 ± 2.1	6.9 ± 0.5
[35]	rNav1.4	I687M/N784K		-54.2 ± 1.7	10.8 ± 1.6	-90.8 ± 1.8	5.6 ± 0.1
[35]	rNav1.4	I687M/F1278I		-40.2 ± 1.5	8.9 ± 1.4	-85.1 ± 4.6	6.4 ± 0.3
[36]	rNav1.4	WT	$\beta1$	-31.4 ± 1.0	9.4 ± 0.8	-73.3 ± 0.1	5.0 ± 0.1
[36]	rNav1.4	3-C	$\beta1$	-14.5 ± 1.2	11.7 ± 1.0	-77.0 ± 0.1	5.2 ± 0.1
[37]	hNav1.5	WT		-53.3 ± 2.7	6.7 ± 0.9	-90.0 ± 2.4	7.1 ± 0.3
[37]	hNav1.5	WT	UVA irradiated	-35.3 ± 4.9	13.8 ± 1.9	-86.8 ± 2.3	12.3 ± 0.7
[38]	rNav1.5	WT		—	—	-72.7 ± 1.1	5.3 ± 0.3
[38]	rNav1.5	WT	ATX-II (10 nM)	—	—	-76.1 ± 1.3	8.2 ± 0.4
[38]	rNav1.5	WT	ATX-II (50 nM)	—	—	-79.1 ± 0.9	7.2 ± 0.4
[38]	rNav1.5	WT	ATX-II (200 nM)	—	—	-84.4 ± 2.1	8.9 ± 0.6
[39]	mNav1.9	WT		-56.1 ± 1.8	5.3 ± 0.2	-71.1 ± 2.8	8.6 ± 1.2
[39]	mNav1.9	WT	PGE2	-64.0 ± 1.8	4.8 ± 0.4	-82.6 ± 2.8	11.3 ± 1.5
[40]	rNav1.8	shRNA	EGFP	-20.4 ± 8.3	6.4 ± 1.9	—	—
[40]	rNav1.8	WT	EGFP	-23.2 ± 2.6	5.3 ± 0.6	—	—
[40]	rNav1.8	WT		-24.1 ± 2.5	4.9 ± 1.1	—	—

[1] McCormick KA, Srinivasan J, White K, Scheuer T, Catterall WA (1999) The extracellular domain of the β1 subunit is both necessary and sufficient for β1-like modulation of sodium channel gating. J Biol Chem 274:32638–32646.

[2] Smith RD, Goldin AL (1998) Functional analysis of the rat i sodium channel in xenopus oocytes. J Neurosci 18:811–820.

[3] Spampanato J, Escayg A, Meisler MH, Goldin AL (2001) Functional effects of two voltage-gated sodium channel mutations that cause generalized epilepsy with febrile seizures plus type 2. J Neurosci 21:7481–7490.

[4] Patton DE, Isom LL, Catterall WA, Goldin AL (1994) The adult rat brain β1 subunit modifies activation and inactivation gating of multiple sodium channel alpha subunits. J Biol Chem 269:17649–17655.

[5] Smith MR, Smith RD, Plummer NW, Meisler MH, Goldman AL (1998) Functional analysis of the mouse scn8a sodium channel. J Neurosci 18:6093–6102.

[6] Ulbricht W (2005) Sodium channel inactivation: molecular determinants and modulation. Physiol Rev 85:1271–1301.

[7] Cummins TR, Aglieco F, Renganathan M, Herzog RI, Dib-Hajj SD, et al. (2001) Nav1.3 sodium channels: rapid repriming and slow closed-state inactivation display quantitative differences after expression in a mammalian cell line and in spinal sensory neurons. J Neurosci 21:5952–5961.

[8] Burbidge SA, Dale TJ, Powell AJ, Whitaker WR, Xie XM, et al. (2002) Molecular cloning, distribution and functional analysis of the na(v)1.6. voltage-gated sodium channel from human brain. Molec Brain Res 103:80–90.

[9] Cummins TR, Dib-Hajj SD, Waxman SG (2004) Electrophysiological properties of mutant nav1.7 sodium channels in a painful inherited neuropathy. J Neurosci 24:8232–8236.

[10] Chen YH, Dale TJ, Romanos MA, Whitaker WR, Xie XM, et al. (2000) Cloning, distribution and functional analysis of the type iii sodium channel from human brain. Eur J Neurosci 12:4281–4289.

[11] Jurkat-Rott K, Mitrovic N, Hang C, Kouzmekine A, Iaizzo P, et al. (2000) Voltage-sensor sodium channel mutations cause hypokalemic periodic paralysis type 2 by enhanced inactiva-

tion and reduced current. Proc Natl Acad Sci 97:9549–9554.

[12] Mohler PJ, Rivolta I, Napolitano C, LeMaillet G, Lambert S, et al. (2004) Nav1.5 e1053k mutation causing brugada syndrome blocks binding to ankyrin-g and expression of nav1.5 on the surface of cardiomyocytes. Proc Natl Acad Sci 101:17533–17538.

[13] Herzog R, Cummins TR, Ghassemi F, Dib-Hajj SD, Waxman SG (2003) Distinct repriming and closed-state inactivation kinetics of nav1.6 and nav1.7 sodium channels in mouse spinal sensory neurons. J Physiol 551:741–750.

[14] Lossin C, Wang DW, Rhodes TH, Vanoye CG, George AL (2002) Molecular basis of an inherited epilepsy. Neuron 34:877–884.

[15] Rhodes TH, Lossin C, Vanoye CG, Wang DW, George AL (2004) Noninactivating voltage-gated sodium channels in severe myoclonic epilepsy of infancy. Proc Natl Acad Sci 101:11147–11152.

[16] Tan HL, Bink-Boelkens MTE, Bezzina CR, Viswanathan PC, Beaufort-Krol GCM, et al. (2001) A sodium-channel mutation causes isolated cardiac conduction disease. Nature 409:1043–1047.

[17] Morgan K, Stevens EB, Shah B, Cox PJ, Dixon AK, et al. (2000) β3: An additional auxiliary subunit of the voltage-sensitive sodium channel that modulates channel gating with distinct kinetics. Proc Natl Acad Sci 97:2308–2313.

[18] Xiao Y, Wright SN, Wang GK, Morgan JP, Leaf A (2000) Coexpression with β1-subunit modifies the kinetics and fatty acid block of hh1(alpha) na(+) channels. Am J Physiol 279:H35–H46.

[19] Xie X, Dale TJ, John VH, Cater HL, Peakman TC, et al. (2001) Electrophysiological and pharmacological properties of the human brain type iia na+ channel expressed in a stable mammalian cell line. Eur J Physiol 441:425–433.

[20] Baker MD, Chandra SY, Ding Y, Waxman SG, Wood JN (2003) Gtp-induced tetrodotoxin-resistant na+ current regulates excitability in mouse and rat small diameter sensory neurones. J Physiol 548:373–382.

[21] O'Leary ME, Digregorio M, Chahine M (2003) Closing and inactivation potentiate the cocaethylene inhibition of cardiac sodium channels by distinct mechanisms. Mol Pharmacol 64:1575–1585.

[22] Vijayaragavan K, Powell AJ, Kinghorn IJ, Chahine M (2004) Role of auxiliary β1-, β2-, and

β3-subunits and their interaction with na(v)1.8 voltage-gated sodium channel. Biochem & Biophys Res Comm 319:531–540.

[23] Chevrier P, Vijayaragavan K, Chahine M (2004) Differential modulation of nav1.7 and nav1.8 peripheral nerve sodium channels by the local anesthetic lidocaine. Brit J Pharmacol 142:576–584.

[24] Vijayaragavan K, Boutjdir M, Chahine M (2003) Modulation of nav1.7 and nav1.8 peripheral nerve sodium channels by protein kinase a and protein kinase c. J Neurophysiol 91:1556–1569.

[25] Coste B, Osorio N, Padilla F, Crest M, Delmas P (2004) Gating and modulation of presumptive nav1.9 channels in enteric and spinal sensory neurons. Mol Cell Neurosci 26:123–134.

[26] Liu C, Dib-Hajj SD, Renganathan M, Cummins T, Waxman SG (2003) Modulation of the cardiac sodium channel nav1.5 by fibroblast growth factor homologous factor 1b. J Biol Chem 278:1029–1036.

[27] Satin J, Kehat I, Caspi O, Huber I, Arbel G, et al. (2004) Mechanism of spontaneous excitability in human embryonic stem cell derived cardiomyocytes. J Physiol 559:479–496.

[28] McNulty MM, Hanck DA (2004) State-dependent mibefradil block of na+ channels. Mol Pharmacol 66:1652–1661.

[29] Wehrens XHT, Rossenbacker T, Jongbloed RJ, Gewillig M, Heidbuchel H, et al. (2003) A novel mutation l619f in the cardiac na+ channel scn5a associated with long-qt syndrome (lqt3): a role for the i-ii linker in inactivation gating. Hum Mut 21:552.

[30] Ferrera L, Moran O (2006) β1-subunit modulates the nav1.4 sodium channel by changing the surface charge. Exp Brain Res 172:139–150.

[31] Filatov GN, Pinter MJ, Rich MM (2005) Resting potential-dependent regulation of the voltage sensitivity of sodium channel gating in rat skeletal muscle in vivo. J Gen Physiol 126:161–172.

[32] Filatov GN, Rich MM (2004) Hyperpolarized shifts in the voltage dependence of fast inactivation of nav1.4 and nav1.5 in a rat model of critical illness myopathy. J Physiol 559:813–820.

[33] Thimmapaya R, Neelands T, Niforatos W, Davis-Taber RA, Choi W, et al. (2005) Distribution and functional characterization of human nav1.3 splice variants. Eur J Neurosci 22:1–9.

[34] Kondratiev A, Tomaselli GF (2003) Altered gating and local anesthetic block mediated by residues in the i-s6 and ii-s6 transmembrane segments of voltage-dependent na+ channels. Mol Pharmacol 64:741–752.

[35] Wang SY, Barile M, Wang GK (2001) A phenylalanine residue at segment d3-s6 in nav1.4

voltage-gated na(+) channels is critical for pyrethroid action. Mol Pharmacol 60:620–628.

[36] Wang G, Wang S (2003) Veratridine block of rat skeletal muscle nav1.4 sodium channels in the inner vestibule. J Physiol 548:667–675.

[37] Wang GK, Wang SY (2002) Modifications of human cardiac sodium channel gating by uva light. J Membrane Biol 189:153–165.

[38] Oliveira JS, Redaelli E, Zaharenko AJ, Cassulini RR, Pimenta KKDC, et al. (2004) Binding specificity of sea anemone toxins to nav 1.1-1.6 sodium channels: unexpected contributions from differences in the iv/s3-s4 outer loop. J Biol Chem 279:33323–33335.

[39] Rush AM, Waxman SG (2004) Pge2 increases the tetrodotoxin-resistant nav1.9 sodium current in mouse drg neurons via g-proteins. Brain Res 1023:264–271.

[40] Mikami M, Yang J (2005) Short hairpin rna-mediated selective knockdown of nav1.8 tetrodotoxin-resistant voltage-gated sodium channel in dorsal root ganglion neurons. Anesthesiology 103:828–836.
